# Transcriptomic analysis of *Bursaphelenchus xylophilus* treated by a potential phytonematicide, punicalagin

**DOI:** 10.21307/jofnem-2020-001

**Published:** 2020-03-18

**Authors:** Qun-Qun Guo, Gui-Cai Du, Ting-Ting Zhang, Mei-Juan Wang, Chao Wang, Hong-Tao Qi, Rong-Gui Li

**Affiliations:** 1 College of Life Sciences, Qingdao University, Qingdao 266071, China; 2 Gaomi People’s Hospital, Weifang 261500, China

**Keywords:** Transcriptomic analysis, Punicalagin, Nematotoxic mechanism, Pine wood nematode, Differentially expressed genes.

## Abstract

Punicalagin showed significant nematotoxic activity against pine wood nematode (PWN), *Bursaphelenchus xylophilus*, in the authors’ previous research. The authors performed high-throughput transcriptomic sequencing of punicalagin-treated nematodes to generate clues for its nematotoxic mechanism of action. The authors identified 2,575 differentially expressed genes, 1,428 of which were up-regulated and 1,147 down-regulated. Based on a comprehensive functional *in silico* analysis, the authors speculate that PWN may respond to the stimulus of punicalagin through phagosome, endocytosis, peroxisome and MAPK signaling pathways. In addition, punicalagin could greatly affect PWN energy metabolism including oxidative phosphorylation. The genes encoding twitchin and a nematode cuticular collagen could be crucial regulation targets of punicalagin, which might contribute to its nematotoxic activity against PWN.

Pine wood nematode (PWN), *Bursaphelenchus xylophilus*, is a plant-feeding nematode parasitizing dozens of pine species and is the cause of pine wilt disease. The disease is highly destructive to pine trees and can spread very quickly from infected trees to reach epidemic proportions (Mamiya, 1983; Mota et al., 1999). Pine wilt disease is primarily controlled using various synthetic nematicides, although their use has increasingly brought concerns on environmental pollution, toxicity to non-target organisms and drug resistance (Kim et al., 2011; Seo et al., 2014; Lee et al., 2017). Thus, ecofriendly natural nematicides derived from plants or microorganisms and their analogs have been considered popular candidates for alternative treatments (Park et al., 2007; Kim et al., 2011; Wang et al., 2012; Seo et al., 2014; Yu et al., 2015; Lee et al., 2017; Huang et al., 2017; Guo et al., 2016, 2017, 2018).

In recent years, we have studied the effects of phytonematicides against *B. xylophilus* (Wang et al., 2012; Guo et al., 2016, 2017, 2018). Consequently, we identified punicalagin from pomegranate (*Punica granatum* L.) rind extract as an active compound against *B. xylophilus*. This compound inhibited the activities of acetylcholinesterase, amylase and cellulase in *B. xylophilus* and caused morphological alterations in the nematodes (Guo et al., 2017). Because punicalagin has the potential to be a low-cost, water-soluble natural nematicide against *B. xylophilus*, its nematotoxic mechanism against *B. xylophilus* is worth further study.

Transcriptomic analysis is essential to interpret the functional elements of the genome and understand development and disease (Wang et al., 2009). Transcriptomic profiling allows the comparison of transcriptomes across a disease state compared to normal cells or of specific experimental stimuli compared to normal physiological conditions. It is thus a potential tool for interpreting the functional elements of the genome and uncovering the biological mechanisms of development and diseases (Wang et al., 2009; Han et al., 2015). In the present study, we performed transcriptomic analysis of *B. xylophilus* after exposure to punicalagin, and we used next-generation sequencing technology to elucidate the mechanism of action of the insecticide.

## Materials and methods

### Reagents and materials

Punicalagin (≥98%, HPLC, pomegranate) was purchased from Sigma-Aldrich (St. Louis, MO, USA). Our strain of *B. xylophilus* was isolated from chips of infected pine wood collected in Nanjing, Jiangsu Province, China using the Baermann funnel technique (Viglierchio and Schmit, 1983). Nematodes were reared on a lawn of *Botrytis cinerea* cultured on potato dextrose agar (PDA) medium in the dark at 26°C for seven days, at which point the ratio of adult male to adult female to juvenile was approximately 1:1:2. An aqueous suspension of nematodes (about 25 nematodes per μl) was prepared and the nematode suspension mixed with punicalagin (500 μM) as the treatment group (P). Treated (P) and normal (CK) nematode samples were cultured in the dark at 26°C for 36 h and then were collected by centrifugation at 4°C for RNA extraction. All samples were assayed in triplicate.

### RNA extraction, library preparation, sequencing and quality control

Total RNA was extracted with TRIzol (Invitrogen, Carlsbad, CA, USA) according to manufacturer’s instructions and was then treated with DNase I (Takara, Dalian, China). RNA quantity and quality were assessed by UV spectroscopy using a NanoDrop spectrophotometer and a Qubit fluorimeter (Thermo Fisher Scientific, Pittsburg, PA, USA), respectively. RNA-Seq transcriptome libraries were constructed using 5 μg total RNA using a TruSeq RNA sample preparation Kit (Illumina, San Diego, CA, USA). mRNAs were isolated using oligo-dT magnetic beads. Fragmentation, cDNA synthesis, end repair, adenylation of 3´ ends, and ligation of the Illumina-indexed adapters were performed according to the manufacturer’s protocol. Libraries were enriched in a 15-cycle PCR reaction and then size-selected using 2% certified Low Range Ultra Agarose (Biorad, Hercules, CA, USA) and quantified using the dye TBS 380 Picogreen (Invitrogen). Clusters were generated by bridge PCR amplification on a cBot System using a TruSeq PE Cluster Kit v3-cBot-HS (Illumina) according to the manufacturer’s instructions. After cluster generation, libraries were sequenced on the Illumina HiSeq 4000 platform (2 × 151 bp read length). To ensure higher accuracy of the successive bioinformatics analysis, clean reads were obtained (Tables A1, A2) by removing reads containing adapters, reads in which more than 10% of the bases were unknown and low quality reads from the raw data using SeqPrep (https://github.com/jstjohn/SeqPrep; -q 20-L 20) and Sickle (https://github.com/ajoshi/sickle; default parameters).

### De novo transcriptome assembly and functional annotation

Transcriptome assembly was carried out using the short-read assembly program Trinity (http://trinityrnaseq.sourceforge.net/, v2013-02-25) using the parameters “--max_memory 50 G --min_kmer_cov 3 --min_contig_length 350 --bfly_opts --V 10” (Haas et al., 2013; Grabherr et al., 2011). Unigenes were compared against those in the NCBI non-redundant protein sequence database and annotated in detail using the BLAST alignment algorithm. Unigenes were annotated by gene ontology (GO, http://www.geneontology.org/) terms describing biological processes, molecular functions and cellular components. BLAST2GO (http://www.blast2go.com/b2ghome) was used to get GO annotations (Conesa and Götz, 2008). The sequences were aligned using the KOG (Eukaryotic Orthologous Groups) database to predict and classify their functions (Grabherr et al., 2011).

Candidate gene pathways were identified and annotated using Kyoto encyclopedia of genes and genomes (KEGG, http://www.genome.jp/kegg/pathway.html) pathway software, KOBAS v. 2.0 (Xie et al., 2011).

### Differentially expressed genes (DEGs)

The expression level for each transcript was calculated using the fragments per kilobase of exon per million mapped reads method to identify DEGs between the two different samples. The program RSEM (http://deweylab.biostat.wisc.edu/rsem/) was used for differential expression analysis (Li and Dewey, 2011). The genes were selected using a false discovery rate (FDR) ≤0.05 and log2(fold-change) ≥1. In addition, functional-enrichment analysis using GO and KEGG was performed to identify which DEGs were significantly enriched in GO terms and metabolic pathways at Bonferroni-corrected from *p* < 0.05 when compared with the whole-transcriptome background. GO functional enrichment and KEGG pathway analysis were carried out using Goatools (https://github.com/tanghaibao/Goatools) and KOBAS (http://kobas.cbi.pku.edu.cn/home.do).

### qRT-PCR validation

The punicalagin-treated (P) and normal (CK) nematode samples were obtained using the same method as those for RNA-Seq. The first-step solution for cDNA synthesis was prepared using 1 μl random hexamers (50 μM), 1 μl dNTPs (at 10 mM each), RNA template (<5 ug), and RNase-free ddH_2_O, topped up to a final solution volume of 10 μl, and incubated at 65°C for 5 min, then cooled in an ice bath. The second-step solution was subsequently prepared using 10 μl the first-step reaction solution, 4 μl 5x PrimeScript II Buffer, 0.5 μl RNase Inhibitor (40 U/μl), 1 μl PrimeScript II RTase (200 U/μl) and RNase-free ddH_2_O topping up to a final solution volume of 20 μl. The final solution were incubated successively at 30°C for 10 min, 42°C for 30 min, and 95°C for 5 min, and it was then cooled in an ice bath.

For validation, we selected six representative unigenes related to basic physiological and metabolic functions of *B. xylophilus*, including: DN8346_c0_g1, encoding dynein heavy cytoplasmic; DN18432_c0_g1, encoding ATP synthase F0 subunit 6 (mitochondrion); DN6713_c0_g1, encoding twitchin; DN14208_c0_g1, encoding small HSP21-like protein; DN8075_c0_g1, encoding a nematode cuticular collagen (collagen triple helix repeat domain containing protein); DN14349_c1_g1, encoding glucosidase 2 subunit beta. A PrimeScript II 1st Strand cDNA Synthesis Kit (Takara, 6210B) was used for first-strand cDNA synthesis experiment. The PCR primers were designed using Primer Premier 5.0 (Table 1). The actin-4 gene of *B. xylophilus* served as an internal control. Quantitative real-time PCR was carried out using SYBR Premix Ex Taq II (Takara). Relative gene expression values was calculated using the 2^−ΔΔC^_T_ method (Livak and Schmittgen, 2001; Revel et al., 2002), and the Pearson correlation coefficient was computed in R.

**Table 1.** Primer sequences used for qRT-PCR validation of differentially expressed genes.

Gene ID	Primer sequence	Amplicon length (bp)
DN8346_c0_g1	Forward: CTGCTGAATGAGTGGGTA	197
	Reverse: AGAAGTTTGAAAGGAGGC	
DN18432_c0_g1	Forward: AAGTGTCACCTCCTTTAC	132
	Reverse: GGTATTCGTTTTGTCCT	
DN6713_c0_g1	Forward: CGAGGTCCGTTAGAAGTG	128
	Reverse: TTGCCAGTCTCAGTGTCC	
DN14208_c0_g1	Forward: GTAAGCCTGGAGAAAAG	195
	Reverse: AGTTGACGGTGTTGGTG	
DN8075_c0_g1	Forward: GCAACCACCAGGAGCAAC	88
	Reverse: CGGAAATGATGGAGAACCC	
DN14349_c1_g1	Forward: AAGTGACGAGCCAGGTA	168
	Reverse: TCACAAACATTCGGACA	
DN6594_c0_g1^a^	Forward: CAACCCCAAGGCTAACA	303
	Reverse: TCACGCACGATTTCACG	

Note: ^a^represents internal control.

## Results

### Transcriptome assembly and functional annotation

**Figure 1:**
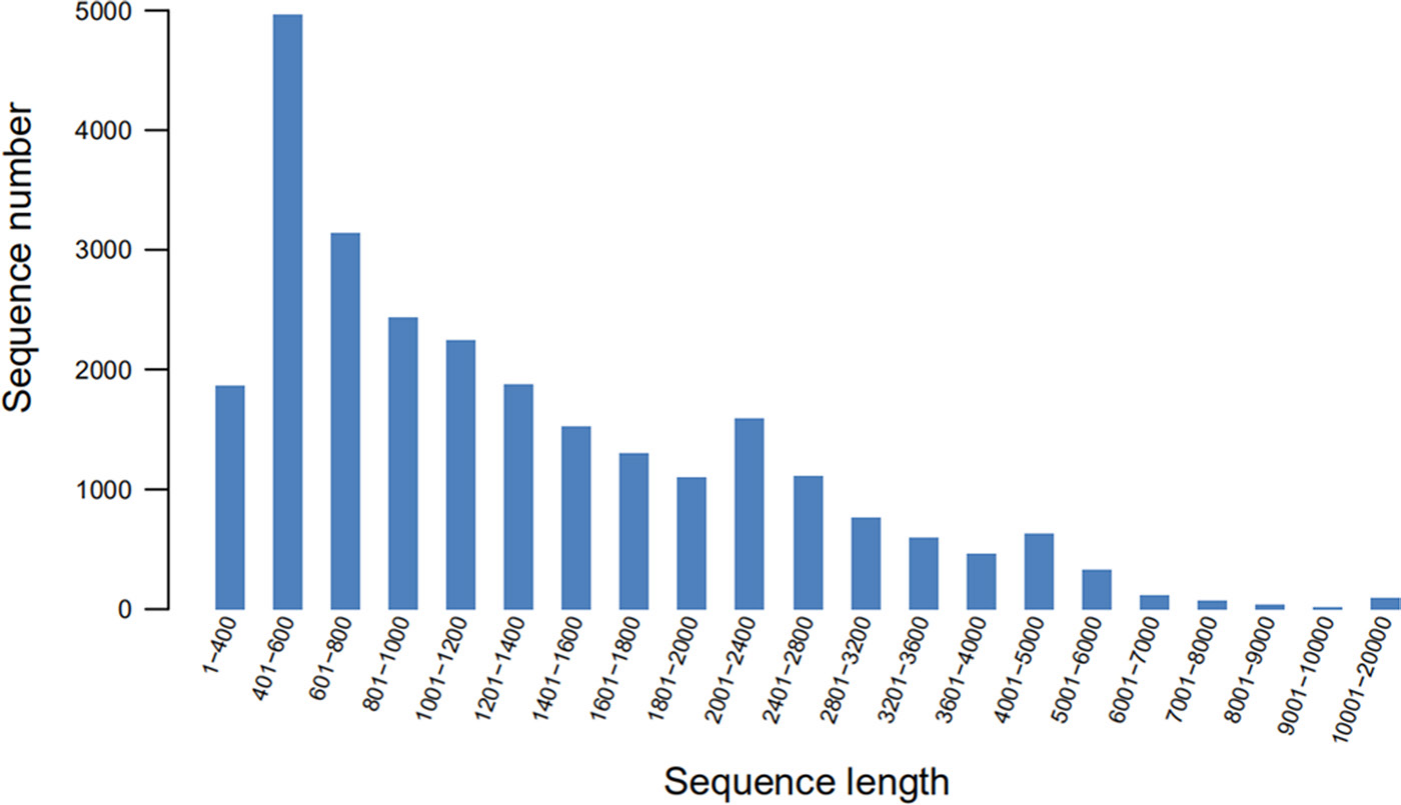
Sequence length distribution of assembled unigenes.

**Figure 2:**
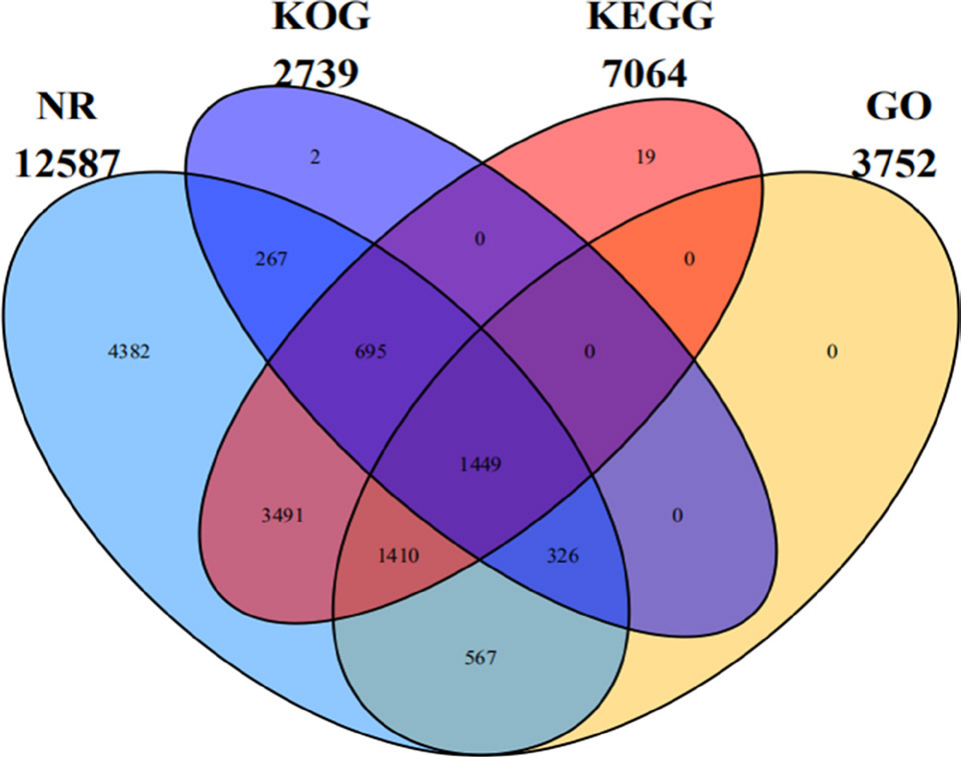
Functional annotation statistics of unigenes.

**Figure 3:**
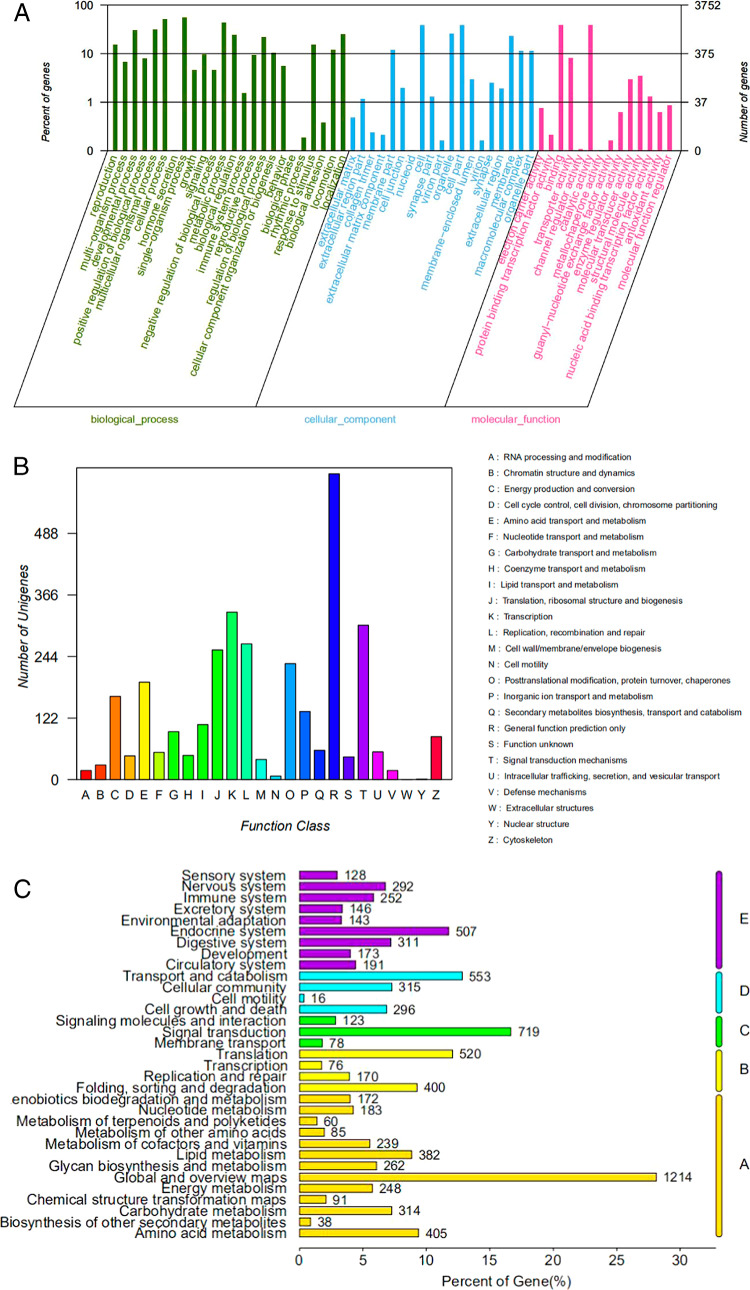
Functional annotation of unigenes. (A) GO functional annotation statistics on level 2. (B) KOG annotation statistics. (C) KEGG pathway annotation statistics. A. Cellular processes. B. Environmental information processing. C. Genetic information processing. D. metabolism. E. Organismal systems.

We identified 21,750 unigenes with a mean length of 1,492 bp and an N50 value of 2,654 bp through de novo transcriptome assembly. The longest unigene was 19,867 bp and the smallest was 351 bp. Unigenes with lengths between 400 and 5,000 bp accounted for approximately 90.29% of the total (Fig. 1). We functionally annotated 12,608 (57.97%) of the unigenes (Fig. 2). The NCBI non-redundant (NR) database contained 12,587 (57.87%) of the genes and 3,752 (17.25%) of these were assigned with the GO terms including biological process, cellular component and molecular function (Fig. 3a). We also classified 2,739 (12.60%) of the genes into 26 functional families (Fig. 3b) and 7,064 (32.48%) were annotated to 321 pathways in five KEGG categories (Fig. 3c).

### Annotation and analysis of DEGs

**Figure 4:**
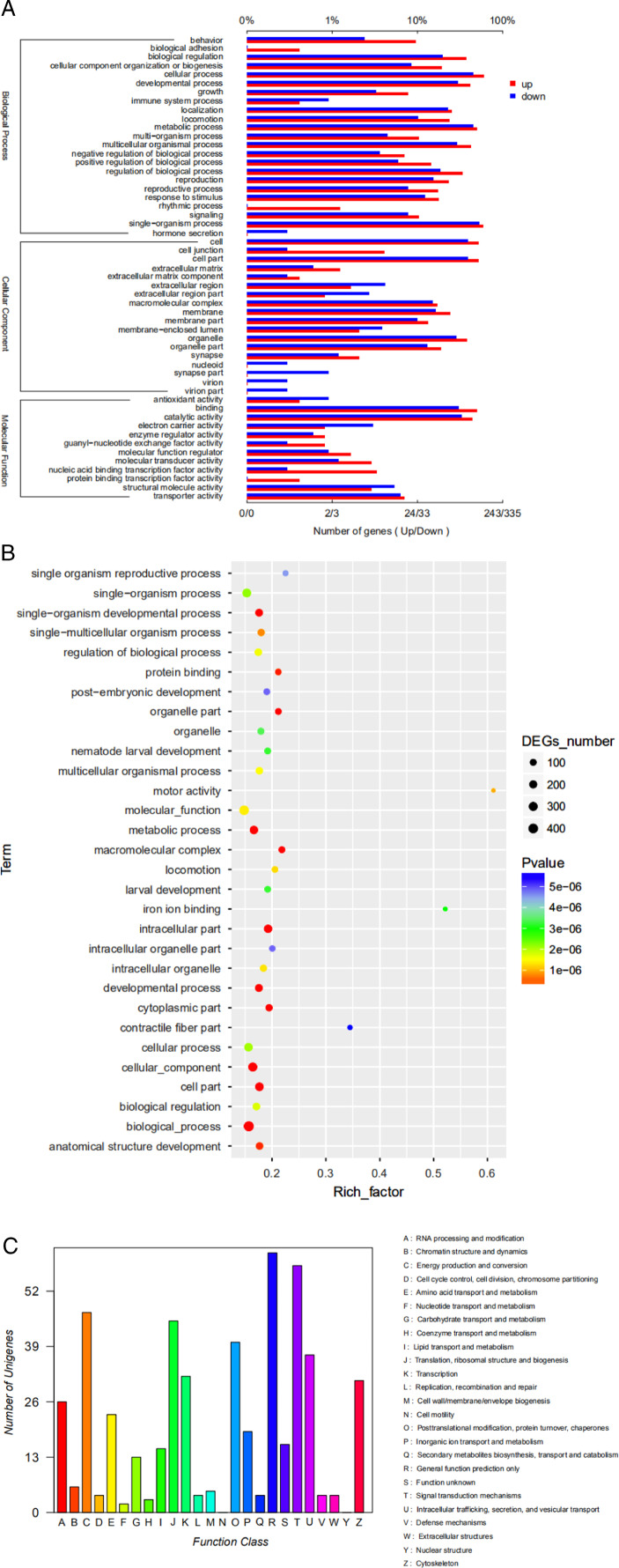
Annotation of differentially expressed genes (DEGs). (A) Column diagram of DEGs using GO annotation. The bottom *x*-axis indicates the number of genes annotated on different GO terms. The *x*-axis indicates the ratios of genes annotated on different GO terms to all terms used for the GO annotation. (B) Scatter diagram of DEGs with GO enrichment. (C) KOG functional classification of DEGs.

We found 2,575 DEGs comprising 1,428 up-regulated and 1,147 down-regulated genes. These genes were related to the basic physiologic and metabolic functions of *B. xylophilus* (Table 2). Our GO functional analysis organized 381 of the DEGs into three groups containing 271, 48 and 62 members that were assigned to 23 categories of biological process, 18 categories of cellular component, and 12 categories of molecular function, respectively. The most highly enriched genes fell into the following categories: those belonging to biological process were single-organism process, cellular process, metabolic process, developmental process, biological regulation, regulation of biological process, localization, locomotion, reproduction and response to stimulus; those belonging to cellular component were cell, cell part, organelle, membrane, organelle part, macromolecular complex and membrane part; those belonging to molecular function were binding and catalytic activity. GO annotation results indicated that punicalagin mainly changed the expression of the genes related to metabolism, development, biological regulation, localization, locomotion and stimulus response that could contribute to its nematotoxic activity (Figs 4a,b).

**Table 2.** Annotated genes differentially expressed in response to punicalagin and related to physiological processes in *Bursaphelenchus xylophilus*.

TRINITY_Gene ID	Annotation	Log_2_ FC	FDR	Type
DN8346_c0_g1	Cytoplasmic dynein heavy chain	3.0314	2.54E-24	Up
DN18432_c0_g1	ATP synthase F0 subunit 6 (mitochondrion)	−3.5432	8.44E-20	Down
DN6713_c0_g1	Twitchin	2.6719	1.19E-18	Up
DN2658_c0_g1	Cytochrome c oxidase subunit I (mitochondrion)	−2.2625	1.66E-12	Down
DN11917_c0_g2	Cytochrome b, partial (mitochondrion)	−2.3332	3.83E-09	Down
DN9703_c0_g1	Cytochrome c oxidase subunit 3 (mitochondrion)	-2.2922	3.88E-09	Down
DN1325_c0_g2	Heat shock protein 20	−2.3758	1.70E-05	Down
DN14208_c0_g1	Small HSP21-like protein	−1.4702	6.73E-05	Down
DN14130_c0_g1	Heat shock protein Hsp-12.2	−1.3353	0.0005945	Down
DN10040_c0_g1	Electron-transfer-flavoprotein	−1.1439	0.01728	Down
DN8075_c0_g1	Nematode cuticle collagen and collagen triple helix repeat domain containing protein	−1.1863	0.01908	Down
DN14349_c1_g1	Glucosidase 2 subunit beta	−1.0567	0.02580	Down
DN22_c0_g1	NADH dehydrogenase subunit 1 (mitochondrion)	2.2809	0.04550	Up

KOG analysis identified 499 DEGs that were annotated to 26 categories (Fig. 4c). The top 10 KOG categories were: general function (61 DEGs), signal transduction (58 DEGs), energy production and conversion (47 DEGs), translation, ribosomal structure and biogenesis (45 DEGs), posttranslational modification, protein turnover, chaperones (40 DEGs), intracellular trafficking, secretion and vesicular transport (37 DEGs), transcription (32 DEGs), cytoskeleton (31 DEGs), RNA processing and modification (26 DEGs), and amino acid transport and metabolism (23 DEGs). Genes related to signal transduction, energy metabolism, translation, ribosomal structure and biogenesis, posttranslational modification, protein turnover and chaperones might be the main nematotoxic targets of punicalagin.

The KEGG annotation aligned the DEGs to 279 pathways. The top 10 pathways that were related to life activities of *B. xylophilus* included oxidative phosphorylation (38 DEGs), endocytosis (35 DEGs), spliceosome (31 DEGs), ribosome (29 DEGs), RNA transport (28 DEGs), carbon metabolism (27 DEGs), ubiquitin mediated proteolysis (24 DEGs), MAPK signaling pathway (24 DEGs), peroxisome (24 DEGs) and biosynthesis of amino acid (22 DEGs).

### Validation of RNA-Seq-based gene expression by qRT-PCR

**Figure 5:**
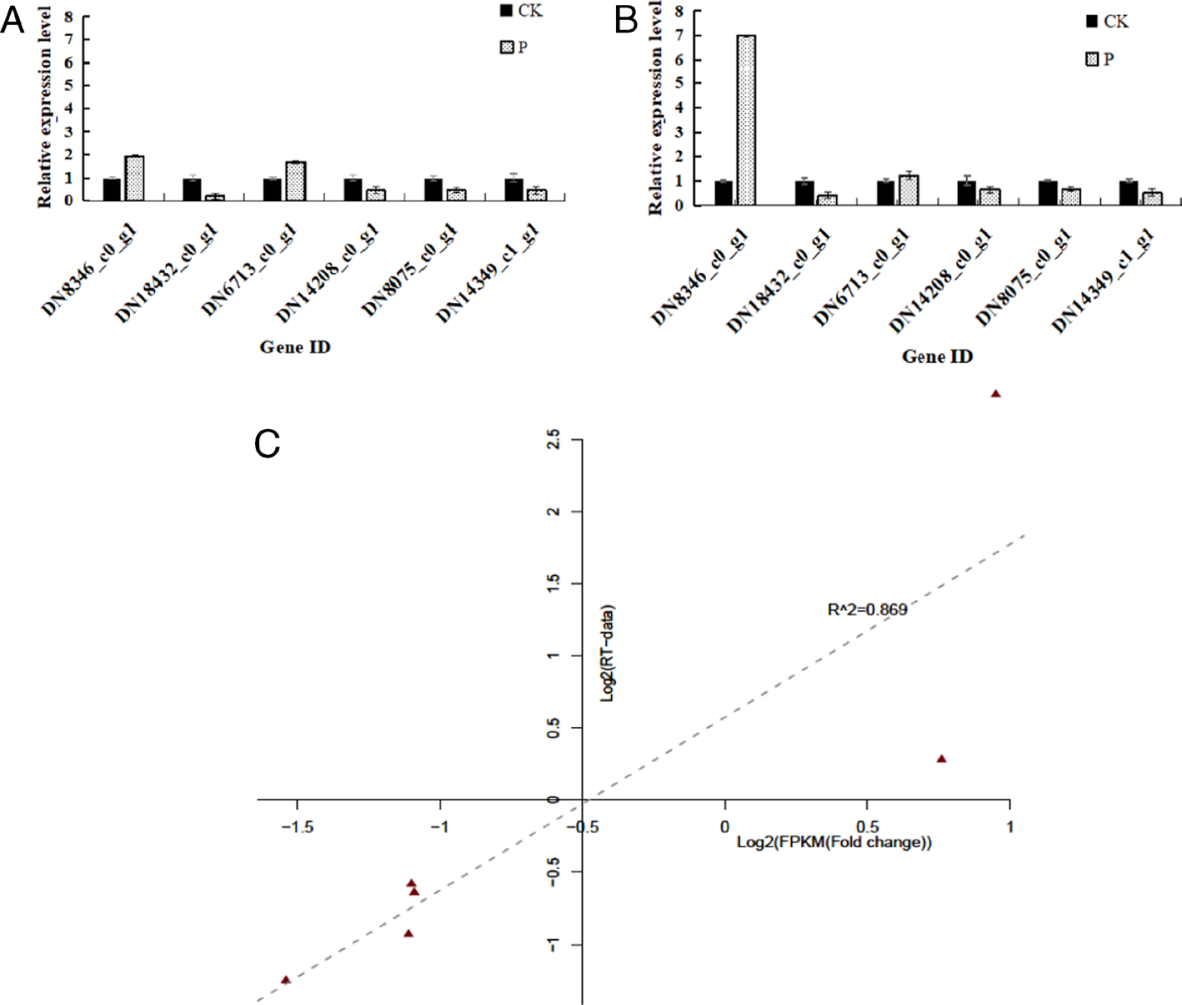
Expression of six differentially expressed genes by (A) RNA-Seq, (B) qRT-PCR and (C) their correlation.

We selected six genes for qRT-PCR analysis to validate the expression profiles obtained by RNA-Seq. The correlation between these two methods was 100% (Fig. 5).

## Discussion

**Figure 6:**
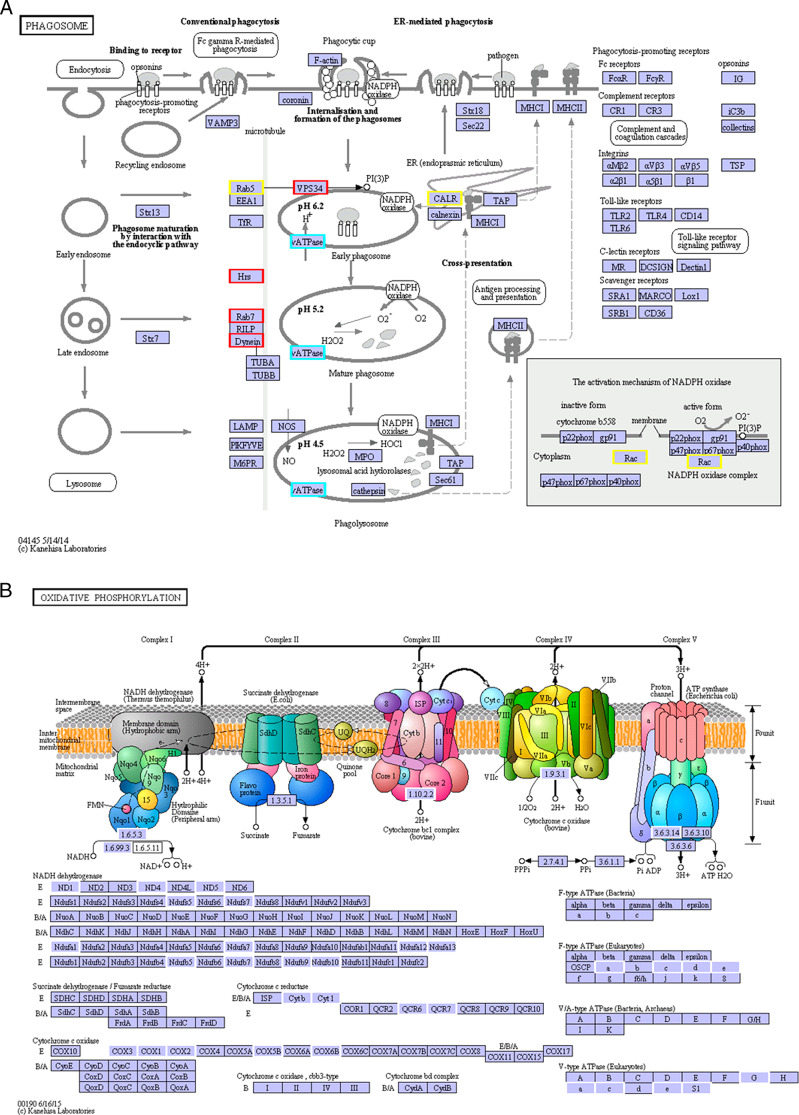
Annotated, enriched KEGG pathway. (A) Annotated KEGG pathway of phagosome. Genes in blue frames with red borders were up-regulated, genes in blue frames with yellow borders were down-regulated, and genes in blue frames with sky-blue borders were simultaneously up-regulated and down-regulated. (B) Annotated KEGG pathway of oxidative phosphorylation about DEGs. Genes in blue frames with white borders were differentially expressed in the pathway.

**Figure A2:**
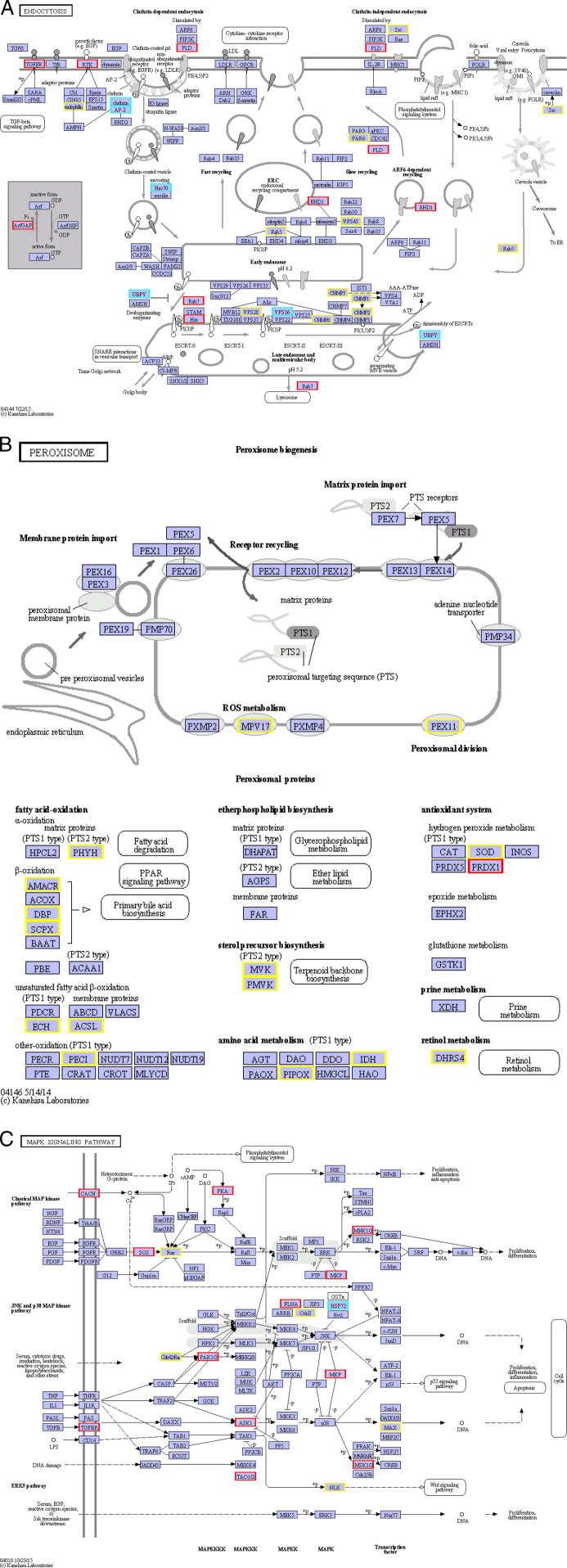
Annotated KEGG pathway of Endocytosis (A), Peroxisome (B) and MAPK signaling pathways (C) about differentially expressed genes. Genes in blue frames with red borders were up-regulated, genes in blue frames with yellow borders were down-regulated and genes in blue frames with sky-blue borders were simultaneously up-regulated and down-regulated.

Through pathway analysis, we identified gene regulation patterns correlated with a response to the nematoxin punicalagin. A significantly up-regulated unigene was that encoding cytoplasmic dynein heavy chain, a putative factor in phagosome maturation of phagosome pathway (Fig. 6a). This protein has been reported to be involved in insulating and decomposing exogenous substances (Rabinovitch, 1995). These clues lead us to speculate that *B. xylophilus* may mount a stress response to punicalagin treatment through the phagosome pathway, with the gene encoding cytoplasmic dynein heavy chain as a possible regulatory target. Other enriched pathways included those of cell stress response, specifically endocytosis, peroxisome and MAPK signaling pathways (Fig. A2), which we hypothesize may likewise contribute to the nematodes’ stress response to punicalagin.

We found that the genes encoding proteins involved in the respiratory electron-transport chain including NADH dehydrogenase, cytochrome c oxidase, cytochrome b and flavoproteins were differently expressed under punicalagin treatment. In addition, KEGG annotation identified additional DEGs involved in oxidative phosphorylation (Fig. 6b), an essential process for basic metabolism (Hatefi, 1985; Gray and Winkler, 1996). Furthermore, energy production and conversion in the KOG database contained the third most DEGs (Fig. 4c). All of these clues indicate that punicalagin may influence the energy metabolism of *B. xylophilus*, which could contribute to the nematotoxic activity of punicalagin.

**Figure A3:**
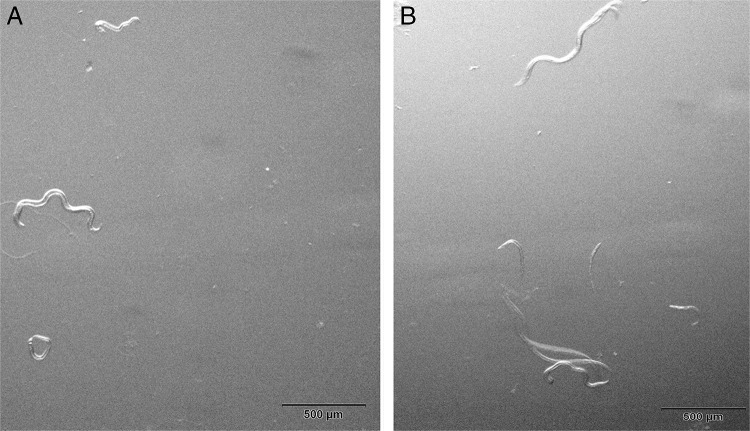
Observation of the normal PWNs (A) and punicalagin-treated PWNs twisting abnormally (B) under microscope.

Lastly, the unigene encoding twitchin was also significantly up-regulated (shown in Table 1). Twitchin is a silk protein related to muscle function in mollusks (Funabara et al., 2007), coinciding with our observation that the PWNs treated with punicalagin under a microscope are abnormally twisted compared with controls (Fig. A3). In addition, the unigene encoding nematode cuticle collagen was down-regulated and this may be related to our previous finding that the body walls of punicalagin-treated nematodes were abnormal (Guo et al., 2017).

In conclusion, we found 2,575 DEGs in total between the two libraries of punicalagin-treated PWNs and control from our comparative transcriptomic analysis. Specifically, we obtained the main DEGs closely related to life activities of PWN, GO terms, KOG calories and KEGG pathways containing more DEGs, and speculated that PWNs could give response to the stimulus from punicalagin through phagosome, endocytosis, peroxisome and MAPK signaling pathways. In addition, punicalagin was supposed to influence PWN energy metabolism. The genes encoding twitchin and nematode cuticular collagen could be crucial regulation targets of punicalagin, which might own to its nematotoxic activity against PWN. Together, these clues may prove valuable for elucidating the nematotoxic mechanism of punicalagin against *B. xylophilus*.

## Figures and Tables

**Figure A1:**
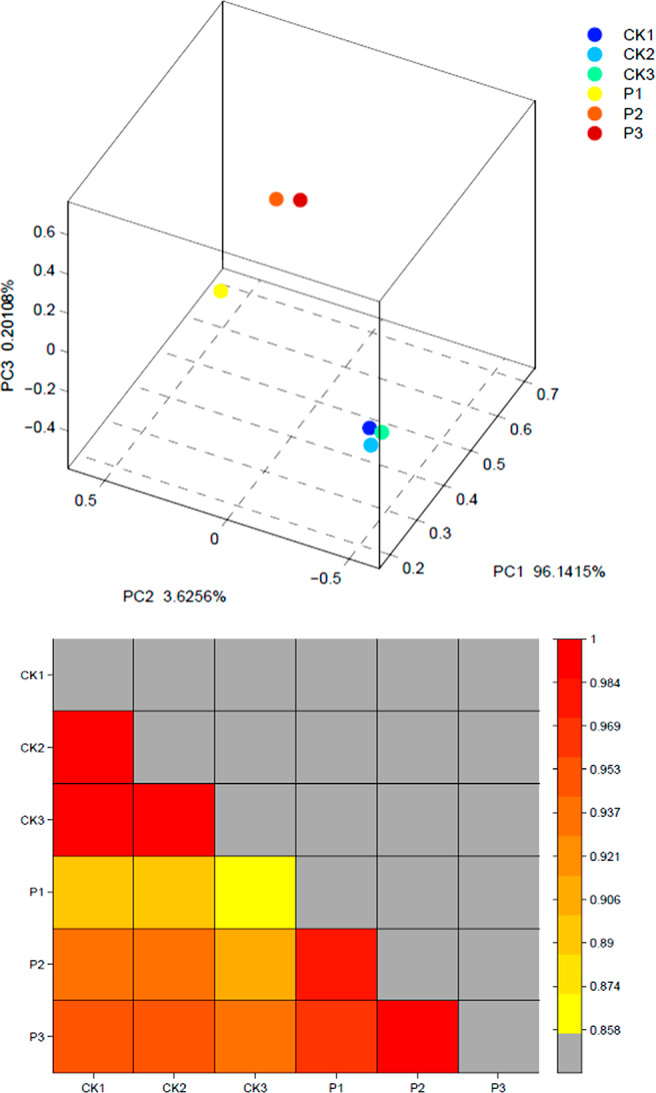
Principal components analysis of variation (A) and correlation coefficient analysis (B) among sequenced transcriptomes to show correlation among samples (control, CK1-3 and the treated samples, P1-3).

## Appendix

**Table A1.** Data statistics of raw reads from six samples for RNAseq.

Sample	Raw reads	Raw reads base (bp)	Q20 (%)	Q30 (%)
CK1	31,003,104	4,650,465,600	95.72	92.01
CK2	37,538,058	5,630,708,700	95.1	91.11
CK3	37,726,236	5,658,935,400	95.31	90.95
P1	35,871,190	5,380,678,500	96.92	93.53
P2	46,131,806	6,919,770,900	96.48	92.26
P3	33,488,164	5,023,224,600	97.32	93.81

Note: CK represents the normal pine wood nematodes, and P represents the punicalagin-treated pine wood nematodes.

**Table A2.** Data statistics of clean reads from six samples after quality control.

Sample	Clean reads	Clean reads base (bp)	Q20 (%)	Q30 (%)
CK1	28,585,924	4,136,450,402	98.48	95.62
CK2	34,085,674	4,903,647,270	98.36	95.36
CK3	34,177,082	4,955,215,491	98.19	94.97
P1	33,854,004	4,938,319,926	98.6	95.91
P2	43,447,298	6,287,507,779	98.11	94.77
P3	32,165,338	4,669,426,049	98.45	95.57
